# Sex‐specific repolarization heterogeneity in mouse left ventricle: Optical mapping combined with mathematical modeling predict the contribution of specific ionic currents

**DOI:** 10.14814/phy2.15670

**Published:** 2023-06-08

**Authors:** S. Erfan Moussavi‐Torshizi, Ehsan Amin, Nikolaj Klöcker

**Affiliations:** ^1^ Institute of Neural and Sensory Physiology Medical Faculty and University Hospital Düsseldorf, Heinrich Heine University Düsseldorf 40225 Düsseldorf Germany; ^2^ Cardiovascular Research Institute Düsseldorf (CARID) Medical Faculty and University Hospital Düsseldorf, Heinrich Heine University Düsseldorf 40225 Düsseldorf Germany

**Keywords:** action potential duration, fast transient outward potassium current, late sodium current, L‐type calcium channel, mathematical modeling, optical mapping, transmural heterogeneity, ultrarapid delayed‐rectifier potassium current

## Abstract

Ventricular repolarization shows notable sex‐specificity, with female sex being associated with longer QT‐intervals in electrocardiography irrespective of the species studied. From a clinical point of view, women are at a greater risk for drug‐induced torsade de pointes and symptomatic long‐QT syndrome. Here, we present an optical mapping (OM) approach to reveal sex‐specific action potential (AP) heterogeneity in a slice preparation of mouse hearts. Left ventricular epicardial repolarization in female versus male mice shows longer and, interindividually, more variable AP duration (APD), yielding a less prominent transmural APD gradient. By combining OM with mathematical modeling, we suggest a significant role of I_Kto,f_ and I_Kur_ in AP broadening in females. Other transmembrane currents, including I_NaL_, only marginally affect basal APD. As in many cardiac pathophysiologies, increasing [Ca^2+^]_i_ poses a risk for arrhythmia, the response of AP morphology to enhanced activation of L‐type calcium channels (LTCC) was assessed in a sex‐selective manner. Both APD and its variation increased significantly more in female versus male mice after pharmacological LTCC activation, which we hypothesize to be due to sex‐specific I_NaL_ expression based on mathematical modeling. Altogether, we demonstrate a more delayed repolarization of LV epicardium, a leveled LV transmural APD gradient, and a more pronounced epicardial APD response to Ca^2+^ influx in females versus males. Mathematical modeling quantifies the relative contributions of selected ionic currents to sex‐specific AP morphology under normal and pathophysiological conditions.

## Graphical abstract

Ventricular repolarization shows sex‐specificity, with female sex being associated with longer QT‐intervals in electrocardiography irrespective of the species studied. Here, we present an optical mapping approach to reveal sex‐specific action potential heterogeneity at high spatial resolution in a slice preparation of mouse hearts. Combining this approach with mathematical modeling allowed for predicting relative contributions of selected ionic currents to action potential morphology under normal and pathophysiological conditions.

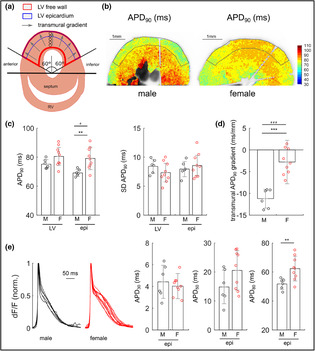

## 1 INTRODUCTION

Clinical and experimental studies have revealed sex‐specific differences in electrocardiography, particularly with respect to ventricular repolarization properties. At baseline, female sex is associated with longer QT intervals (Abi‐Gerges et al., 2004; Locati et al., 1998). Particularly notable become such differences when comparing the prevalence of cardiac arrhythmias between sexes. Women are significantly more prone to symptomatic Long‐QT Syndrome (LQTS) and Torsade de Pointes (TdP) tachycardia than men (Ebert et al., 1998; Makkar, 1993). It is widely assumed that differences in ion channel expression and function account for the sex‐specific differences in phenotype. However, many of the previous studies on sex‐related variations in murine left ventricular ionic current expression and function have reported conflicting findings (Table 1). Crucial repolarizing currents such as the fast transient outward K^+^ current (I_Kto,f_) and the ultrarapid delayed‐rectifier K^+^ current (I_Kur_) have been found mutually up‐ and down‐regulated or not regulated at all in female versus male mice (Brunet et al., 2004; Trépanier‐Boulay et al., 2001; Wu & Anderson, 2002). Attempts to interpret such inconsistencies attributed ionic current regulations to varying sex hormone levels. Androgens have been shown to enhance I_Kur_ density and subsequently shorten action potential duration (APD) in male mice (Brouillette et al., 2005), whereas high levels of estrogen may down‐regulate I_Kto,f_ and I_Kur_ densities prolonging the APD in female mice (Trépanier‐Boulay et al., 2001). Also, an elevated late sodium current (I_NaL_) component in the AP may increase the susceptibility of female mice to LQT‐related arrhythmias (Wu & Anderson, 2002). Whether the increased I_NaL_ function, however, simply reflects a decreased repolarization reserve due to down‐regulation of K^+^ channels remains elusive.

**TABLE 1:** Semi‐quantitative comparison of previously reported ion channel gene and functional current expression in female versus male mice of indicated strains.

Strain	Location	Ionic current	Method	Age	Reference
CD1	RV/LV (Epi)	I_Kur_ (↓) I_to,f_ (≈) I_to_ (≈) I_Kss_ (≈) I_Ks_ (≈) I_K1_ (≈) I_Ca,L_ (≈) I_Na(peak)_(≈)	Isolated cardiomyocyte (patch‐clamp, RNase Protection Assay)	2–3 months	(Trépanier‐Boulay et al., 2001)
C57BL/6 H/H	LV	I_NaL_ (↑) I_Na_ (≈)	Isolated cardiomyocyte (patch‐clamp)	‐	(Lowe et al., 2012)
C57BL/6	RV	I_K,total_ (↓) I_Kur_ (↓) I_Ks_ (≈) I_K1_ (≈) I_Kss_ (↓)	Isolated cardiomyocyte (patch‐clamp, RT‐PCR)	‐	(Saito et al., 2009)
C57BL/6	LV	I_kur_ (↑) I_to_ (↓)	Isolated cardiomyocyte (patch‐clamp)	10–12 months	(Wu & Anderson, 2002)
C57BL/6	septum	I_K,total_ (↓)	Isolated cardiomyocyte (Patch‐clamp, RT‐PCR)	10 months	(Crump et al., 2016)
C57BL6, FVB, Sv129	RV/LV (Epi, Endo)	I_to,f_ (≈) I_Kur_ (≈) I_Kss_ (≈) I_K1_(≈)	Isolated cardiomyocyte (patch‐clamp, Western blot)	2–3 months	(Brunet et al., 2004)
C57BL6, FVB, Sv129, CD1	septum	I_to,f_ (↓) I_to_ (↓) I_Kur_ (↓) I_Kss_ (↓) I_K1_(≈)	Isolated cardiomyocyte (patch‐clamp, Western blot)	2–3 months	(Brunet et al., 2004)
CD1	LV	I_Kur_ (↓) I_to,f_ (≈) I_Kss_ (≈)	Isolated cardiomyocyte (patch‐clamp, Western blot)	2–3 months	(Brunet et al., 2004)

Abbreviations: I_K,total_, total outward K^+^ current; I_K1_, inward rectifier K^+^current; I_Kss_, the steady‐state K^+^ current; I_Kur_, ultrarapid delayed rectifier K^+^ current; I_Na_, inward Na^+^ current; I_NaL,_ late inward Na^+^ current; I_sus_, sustained outward K^+^ current; I_to,f_, fast transient outward K^+^ current; I_to,s_, slow transient outward K^+^ current.

The majority of experimental research in this field exploited models of dissociated cardiomyocytes, particularly useful for combining single‐cell electrophysiology with molecular analysis. However, cell isolation entails noteworthy drawbacks. Not only will the regional origin of the studied cells get at least partially lost, but also will enzymatic digestion harm the extracellular matrix and hence important ion channel modulators or even accessory subunits (Kline & Mohler, 2014; Liu & Melchert, 2010; Salvage et al., 2020). In the present study, we therefore established an optical mapping approach to visualize transmembrane voltage in thin slices of murine ventricular tissue offering accurate locoregional AP features. We sought out to investigate the APD and its regional intra‐ and interindividual variability in the left ventricle of adult male and female mice, both under baseline conditions and in response to pharmacological calcium channel activation resembling a cardiac stress factor. Mathematical modeling approximated the respective contribution of selected ionic currents on AP morphology.

## 2 MATERIALS AND METHODS

### 2.1 Animals

All animal experiments comply with the EU Directive 2010/63/EU on the protection of animals used for scientific purposes. Studies were approved by the local Animal Care and Use Committee of the University of Düsseldorf (# O7/11) and the Animal Ethics Committee of the North Rhine‐Westphalia Nature, Environment and Consumer Protection Agency (LANUV). Adult male and female C57BL/6J mice (20–30 g; 22–27 weeks; Janvier Labs), maintained at a 12/12 h light/dark cycle and fed ad libitum water and chow diet were used for the study. All mouse experiments were performed in the same time window of the day (±2 h) to control for putative heterogeneities in cardiac electropysiology caused by circardian rhythm.

### 2.2 Tissue slice preparation

Mice were injected intraperitoneal with 300 IU/kg heparin 15 min prior to heart harvesting. Animals were then sedated with 2.5% (v/v) isoflurane inhalation and sacrificed by cervical dislocation. Hearts were rapidly excised and placed in ice‐cold oxygenated (95% O_2_, 5% CO_2_) bicarbonate‐buffered extracellular solution (containing in mM: 123 NaCl, 1.8 CaCl_2_, 5.4 KCl, 1.2 MgCl_2_, 1.4 NaH_2_PO_4_, 24 NaHCO_3_, and 10 glucose). The aorta was immediately perfused retrograde by the same solution via a peristaltic pump (flow rate of 1.5 mL/min) at room temperature. To ensure that isolated hearts beat rhythmically, the perfusion continued for at least 3 min. Perfusion was subsequently changed to bicarbonate‐buffered extracellular solution supplemented with the potentiometric dye Di‐8‐ANEPPS at 3 μM (D3167, Invitrogen). Following dye loading for 4 min, the hearts were perfused with 10 μM (−)‐blebbistatin (S7099, Selleckchem) dissolved in bicarbonate‐buffered extracellular solution until the heart ceased beating (~5 min).

After dissecting atria and major vessels, ventricles were embedded in 4% low‐melt agarose (Art. No. 6351.2, Carl Roth) dissolved in phosphate buffered saline at 37°C. The agarose blocks were chilled quickly until complete solidification. The agarose‐embedded ventricles were glued with tissue adhesive histoacryl (175,182, Bbraun) on a specimen holder and were sectioned transversally into 350 μm thick slices employing a vibratome (LEICA VT1200S) with steel blades at a progression speed of 0.03 mm/s and lateral blade vibration amplitude of 2 mm (Wang et al., 2015). During the sectioning process, the tissues were kept in an ice‐cold oxygenated bicarbonate‐buffered extracellular solution (95% O_2_, 5% CO_2_) containing 10 μM (−)‐blebbistatin.

Immediately after sectioning, the slice was transferred into a custom‐made circulation chamber filled with 10 μM (−)‐blebbistatin containing bicarbonate‐buffered solution. By positioning a glass spiral before the chamber entrance and regulating the solution flow rate, the temperature of the chamber was kept at 35°C. The slices were stimulated using a unipolar platinum‐iridium electrode (ThermoFisher) connected to an electrical stimulator (STG4002, Multi Channel Systems MCS GmbH) with a cycle length of 200 ms, a pulse width of 1 ms, and 1.5 times of threshold strength (3–5 V). For the cardiac slices to reach an electrophysiological steady state, they had to be continuously paced for at least 30 min. During this time, the signal‐to‐noise ratio of fluorescence intensity likewise increased and reached a stable state. All short‐axis slices included in this study were taken from the middle one third of the heart to control for AP heterogeneity over the longitudinal axis (base/apex).

### 2.3 Optical mapping

To excite Di‐8‐ANNEPS, an LED light source (LEX3‐G; 525 nm; SciMedia/Brainvision) connected to a THT Macroscope (SciMedia/Brainvision) was used. The light passed through an excitation filter (BP531 nm/40) was deflected by a dichroic mirror (580‐FDI) towards the perfusion chamber and focused onto the slice. The fluorescent signals emitted by the slice were gathered via a 1.0X objective (LEICA) and filtered using a long‐pass filter (LP600 nm). The filtered fluorescent emission was captured by a MiCAM05‐N256 imaging system (SciMedia/Brainvision) equipped with a CMOS image sensor (mapping field area: 10 × 10 mm (256 × 256 pixels); frame rates: 1 kHz; SciMedia/Brainvision). The effective spatial resolution is 50 μM/pixel. The proprietary BV Workbench (Brainvision) was used to operate the optical recordings and to store them in the appropriate data format before being submitted to customized MATLAB analysis.

### 2.4 FPL64176 application

After APD recordings had reached a steady‐state, FPL64176 (250 nM) was added to the circulating extracellular solution, while APs were continuously monitored in time intervals of 5 min until FPL64176‐induced prolongation of APD again reached steady‐state.

### 2.5 Analysis of optical data

Data processing, analysis, and visualization were carried out applying self‐developed MATLAB scripts. Processing of optical data comprised ensemble averaging, drift correction by polynomial fitting, and spatial mean filters of 3 × 3 pixels. The data processing was partially inspired by RHYTHM source code (Gloschat et al., 2018). The data were analyzed by extracting activation times and AP duration at 30%, 50%, 80%, and 90% of repolarization (APD_30_, APD_50_, APD_80,_ and APD_90_). In order to investigate APDs, we adjusted the “*findpeaks*” function to identify local maxima and widths at various peak amplitude levels. The vector drawing program Inkscape was used to rearrange and edit MATLAB plots and maps.

### 2.6 Mathematical modeling

Simulations were created using a dynamic mathematical model of a mouse ventricular AP (Li et al., 2010). The predominant transmembrane currents, ionic pumps, and exchangers are included into the model. Also, intracellular Ca^2+^ homeostasis formulations are taken into account. OpenCARP environment has been used to implement the simulations (Plank et al., 2021). The AP traces were resampled to 1 ms time intervals resembling the optical mapping temporal resolution.

### 2.7 Statistical analysis

Data were reported as mean ± standard deviation, and statistical analyses using Student's *t*‐tests determined statistical significance reported as: *p < 0.05, **p < 0.01, and ***p < 0.001. When several slices from an individual animal were investigated, hierarchical, nested statistics were applied to test for significant differences (Eisner, 2021; Sikkel et al., 2017). Coefficient of variability statistics was used to estimate the spread of APDs and its gradients in males versus females, and the signed‐likelihood ratio test (SLRT) was used to examine their significance (Krishnamoorthy & Lee, 2014). We used the R package “cvequality” to test for significant differences.

## 3 RESULTS

### 3.1 Sex‐specific repolarization properties in mouse heart slices

**FIGURE 1:**
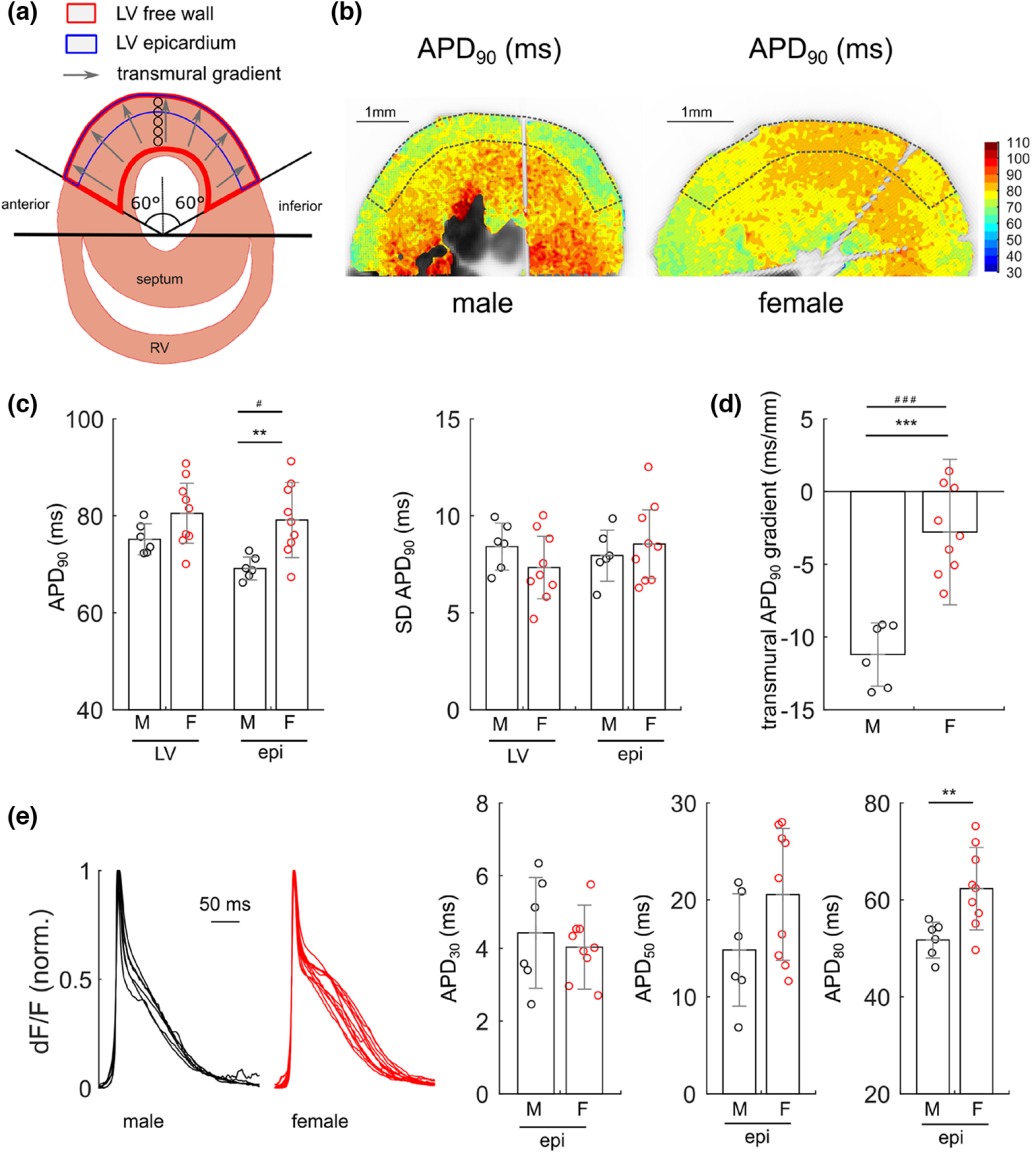
Optical mapping allows for sex‐specific comparison of the left ventricular transmural mouse action potential. (a) Schematic illustration of the areas of interest analyzed in the left ventricle (LV). An appropriate angle was chosen to exclude anterior and inferior heart regions. Whole LV free wall, epicardium (one third of the LV free wall), and the transmural gradient of action potential duration (APD) were investigated parameters. Papillary muscles were explicitly excluded from LV wall analysis. (b) Optical mapping of APD at 90% repolarization (APD_90_) in a representative male (left panel) and female (right panel) LV short‐axis heart slice, respectively. (c) Bar graphs showing APD_90_ and its standard deviations (SD), calculated from whole LV free wall (LV) and epicardium (epi) of slices from male (M) and female (F) mice (M: *n* = 6, F: *n* = 9; **p = 0.0061; ^#^MSLRT = 5.1, *p* = 0.023). (d) Quantification of transmural gradient vectors of APD_90_ (M: *n* = 6, F: *n* = 9; ***p = 0.000018; ^###^MSLRT = 18.4, *p* = 1.7 × 10^−5^) (e) Overlay of male and female epicardial APs (regions defined in b by dashed lines), respectively, and quantification of the APD at indicated % repolarization (*n* = 6 slices from *N* = 6 male mice, *n* = 12 slices from *N* = 9 female mice; APD_80_; **p = 0.009).

Cardiac slice preparations have been developed for systematic optical analyses of regional gradients of transmembrane voltage and [Ca^2+^]_i_ across the ventricles (Ripplinger, 2018; Wang et al., 2015; Wen et al., 2018). First, we optimized the optical system and sample preparation, increasing both temporal and spatial resolution and signal quality (see Methods). For optical analysis of locoregional AP features in adult male and female mice, we focused on three parameters of interest within the LV: (i) the whole LV free wall, (ii) epicardium (outer one third of the LV free wall), and (iii) the LV transmural gradient of APD (Figure 1a). Color‐coded maps reveal a sex‐specific spatial distribution of APD at 90% repolarization (APD_90_) across the LV at a stimulation frequency of 5 Hz (Figure 1b). As quantified in Figure 1c, epicardial APD_90_ was significantly greater in LV slices of females than of males (*p* = 0.0061, *n* = 6 male and *n* = 9 female mice, respectively), whereas neither the overall LV APD_90_ nor their standard deviations (SD) differed between sexes. Instead, a strong negative transmural gradient of APD_90_ towards epicardium was observed in slices of male mice, which was significantly reduced in slices of female mice (Figure 1d; Table S1, *p* = 0.00002). In addition, analyses of coefficients of variations (COV) showed significantly increased dispersion of epicardial APD_90_ (MSLRT = 5.1, *p* = 0.023) and of transmural APD gradients (MSLRT = 18.4, *p* = 1.7 × 10^−5^) in female versus male mice.

With the most robust sex‐specific APD heterogeneity apparently occurring in epicardium, we focused our further investigation specifically on this area of interest. Overlaying epicardial AP morphologies disguises longer APD, particularly at late repolarization, in female over male mice, as depicted and quantified in Figure 1e. For quality control, it should be mentioned that the absolute APD_90_ in male LV was 69 ± 3 ms, which is in perfect agreement with the values recorded from intact mouse heart at this pacing rate (Table 2) (Knollmann et al., 2001; Saito et al., 2005).

**TABLE 2:** Sex‐specific comparison of action potential morphology (M: male, F: female).

Strain	Location	APD_20_ (ms)		APD_50_ (ms)		APD_90_ (ms)		Stimulation frequency (Hz)	Reference
		M	F	M	F	M	F		
CD1	V	2.7 ± 0.2	4.2 ± 0.4	4.4 ± 0.3	9.2 ± 1.3	14.8 ± 1	22.7 ± 2.1	4	(Trépanier‐Boulay et al., 2001)
C57BL/6 H/H	V	‐	‐	6.2 + 0.5	9.3 + 0.8	23.3+ 1.8	35.2 + 2.7	2	(Lowe et al., 2012)
C57BL/6	RV	‐	‐	3.11 ± 0.2	5.21 ± 0.8	12.52 ± 1.2	29.53 ± 6.2	‐	(Saito et al., 2009)
C57BL/6	LV	‐	‐	11 ± 2 8 ± 2	12 ± 2 15 ± 3	47 ± 7 42 ± 9	54 ± 5 63 ± 6	0.3, 1	(Wu & Anderson, 2002)
C57BL/6	septum	‐	‐	3.7 ± 0.3	5.7 ± 0.9	29.7 ± 5.2	30.7 ± 4.5	1, 3	(Brunet et al., 2004)
CD1	V	3 ± n.r.	‐	4 ± n.r.	‐	12.7 ± 1.2	‐	1	(Brouillette et al., 2004)
C57BL/6	V	3.1 ± 0.1	‐	9.0 ± 0.6	‐	37.1 ± 2.7	‐	1	(Chan et al., 2011)
C57BL/6	LV	‐	‐	‐	‐	56.8 ± 7.3	‐	1	(Coppini et al., 2017)
C57BL/6	RV	‐	‐	‐	‐	79.3 ± 0.6	‐	1	(Ferrantini et al., 2016)
ICR	LV tissue	‐	‐	5.9 ± 1.7	‐	49 ± 12 (69 ± 9.7)^a^	‐	5–9	(Knollmann et al., 2001)
C57BL/6	LV	‐	‐	‐	‐	36 ± 3	‐	1	(London et al., 2001)
C57BL/6	LV tissue	‐	‐	‐	‐	67.4 ± 2.7	‐	5	(Saito et al., 2005)

Abbreviations: n.r., not reported; V, ventricles.

^a^ After contractile uncoupling.

### 3.2 Simulation of ionic current contribution to the mouse cardiomyocyte AP morphology

**FIGURE 2:**
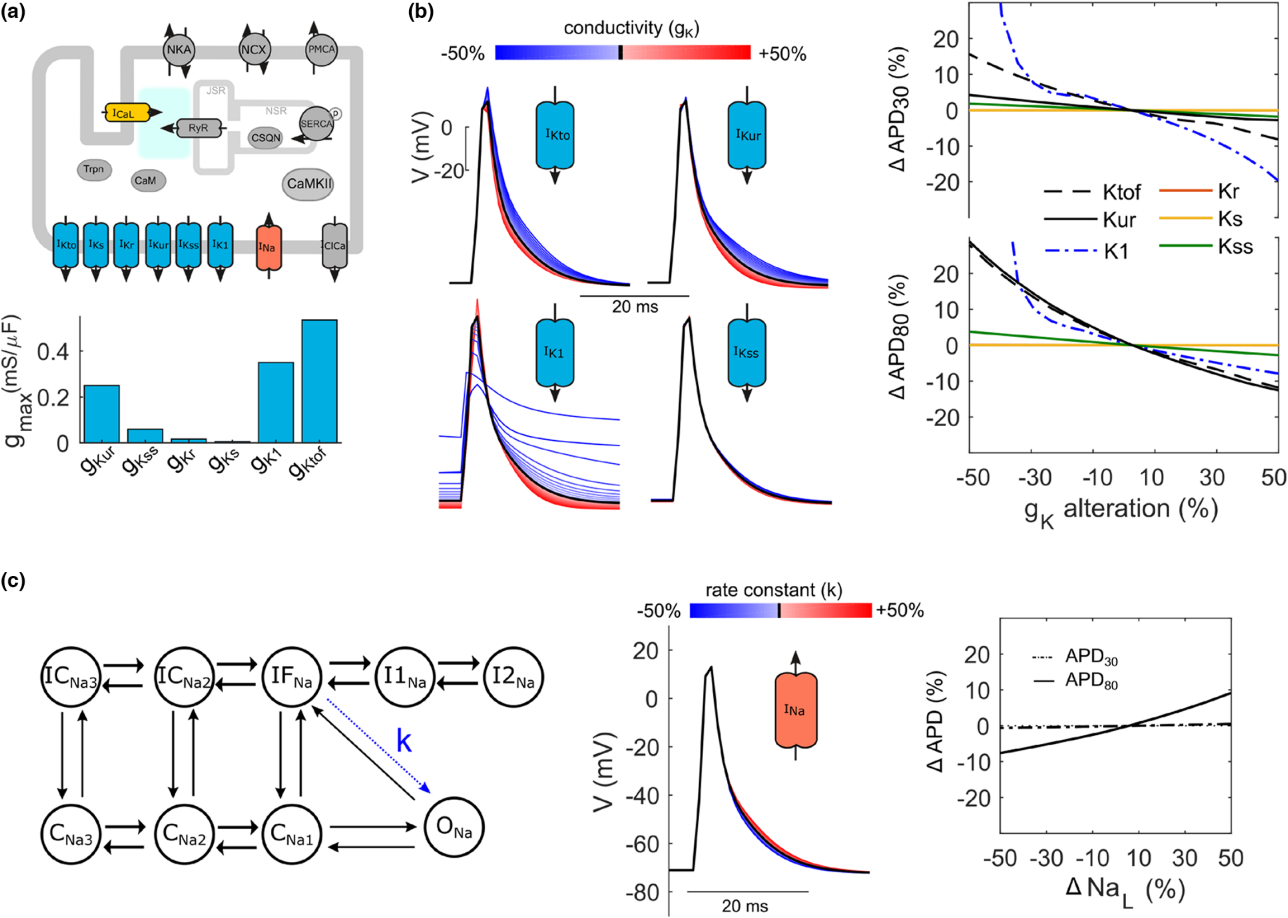
Mathematical modeling of the mouse action potential upon alteration of ion channel conductivities contributing to the repolarization reserve. (a) Schematic diagram of a model for a single‐cell AP in left ventricular mouse cardiomyocytes comprising indicated ionic currents and calcium fluxes (upper panel). Relative contribution of indicated K^+^ conductivities implemented in the model (lower panel). (b) Simulation of APs under the gradual increase and decrease (5% per trace) in indicated K^+^ conductivities from control to ±50% (left) and corresponding changes in APD_30_ and APD_80_ (right). (c) State diagram of a Markov model for the Na_v_ channel (left). C_Na1_, C_Na2_, and C_Na3_ resemble closed states; O_Na_ resembles an open state; IF_Na_ resembles a fast‐inactivated state; I1_Na_ and I2_Na_ resemble intermediate inactivated states; and IC_Na2_ and IC_Na3_ resemble inactivated closed states. The effect of modifying the rate constant *k* for channel re‐opening from IF_Na_ is simulated during AP generation and APDs are quantified by plotting their relative change at indicated % of late sodium current.

Mathematical models have been an essential tool for understanding and exploring the complex interactions of ionic currents and their contribution to AP generation and morphology. The evident controversy on sex‐specific ion channel expression and function (Table 1) prompted us to exploit mathematical modeling in order to contrast and compare the effects of putative differential ionic current modulation in more detail. Figure 2a shows a schematic diagram of an electrophysiological model of a single mouse cardiomyocyte established and further optimized by the Bondarenko group (Bondarenko, 2004). We have implemented the formulations in openCARP (Plank et al., 2021). As most debates on sex‐specific ion channel regulation in cardiomyocytes are about potassium channels, we first compared the maximum conductivities of potassium currents implemented in the model (Figure 2a, lower panel). It shows that in the mouse cardiomyocytes, g_Kr_ and g_Ks_ play rather minor roles compared to others, for which we excluded them from further analysis. Instead, the influence of the remaining K^+^ currents on AP morphology was assessed in more detail by mathematically simulating respective increases or decreases in current densities of up to ±50%. A stimulus pulse was applied for a period of time of 5 s at a frequency of 5 Hz. We focused on changes in AP morphology induced by the trains of simulation while conductivity varied and overlayed the respective last APs of two stimulations to better appreciate the differences (Figure 2b). As expected, a reduction in all K^+^ current densities prolonged the APD and solely changes in I_K1_, altered the resting membrane potential. As the latter, however, did not show any reliable sex‐specific difference in previous experimental data (suppl. Figure 1), we further excluded I_K1_ from investigation in this context. A decrease in I_Kto_ was predicted to prolong APD_30_ and APD_80_ by up to 16% and 28%, respectively. For a reduction in I_Kur_, the model suggested an increase in APD up to 4% and 29% at early and late repolarization phases, respectively. Finally, for decreases in I_Kss_, the model indicated APD changes below 4%. These findings are summarized in Figure 2b (right panel), plotting APD_30_ and APD_80_ as a function of respective conductivity (g): Whereas both I_Kto_ and I_Kur_ have a similarly strong impact on late repolarization (APD_80_), early repolarization (APD_30_) is predominantly shaped by only I_Kto_. Based on literature (Lowe et al., 2012), we finally aimed at simulating also the effects of the basal late sodium current on AP morphology. Given the Markov model for I_Na_ depicted in Figure 2b, the rate constant of channel transition from a fast‐inactivated state (IF_Na_) to the open state (O_Na_) was modulated by ±50%. The model yielded a rather moderate increase in APD_80_ by 9% upon a 50% increase in the rate of re‐opening. Altogether, the mathematical simulations point to a predominant influence of I_Kto_ on early repolarization, whereas both I_Kto_ and I_Kur_ share a similar and dominant contribution to late repolarization, partially counteracted by basal I_NaL_.

### 3.3 L‐type calcium channel (LTCC) activation disguises sex‐dependent repolarization reserve

**FIGURE 3:**
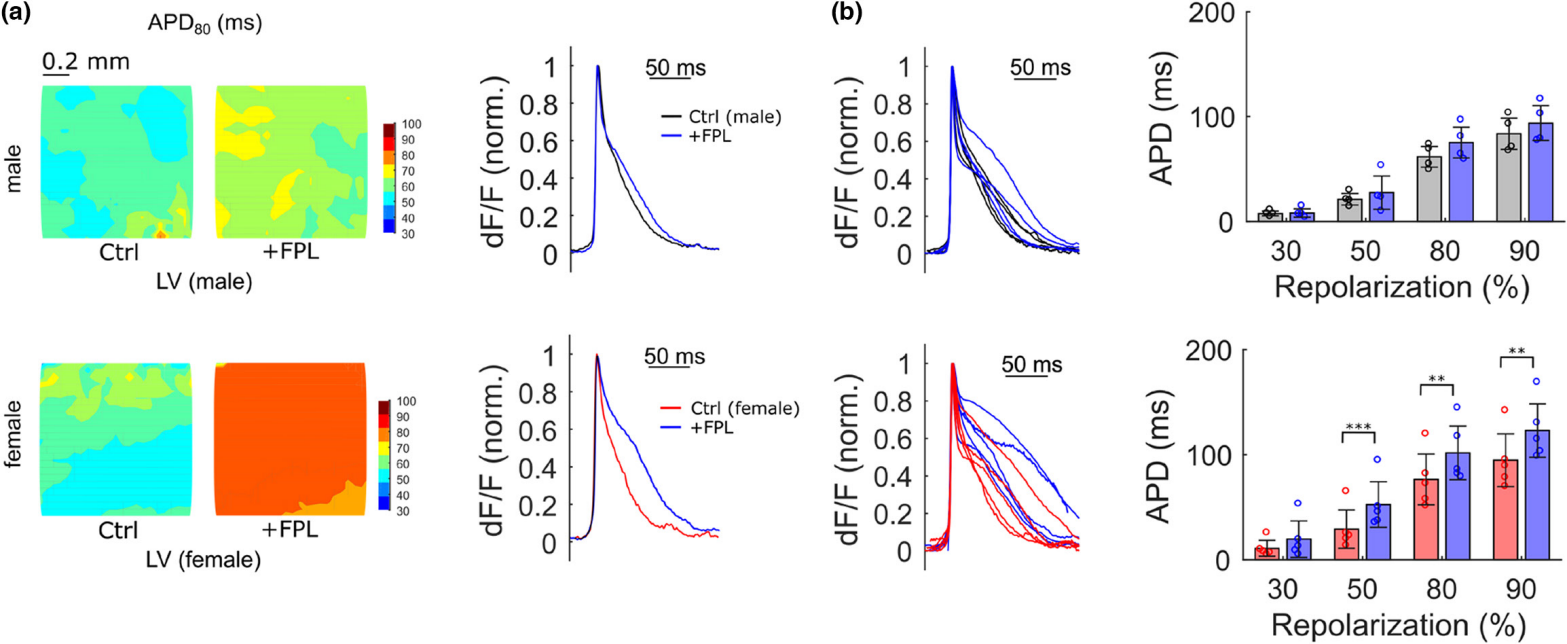
Sex‐specific increase in action potential duration upon L‐type calcium channel activation. (a) Optical mapping of APD_80_ in a representative region of the left ventricle (LV) before and after the application of the pharmacological calcium channel activator FPL 64176 (250 nM) in male (upper) and female (lower) heart slice preparations. Two representative APs averaged from indicated areas before and after application of FPL 64176 are overlaid (right). (b) Overlay of male and female LV APs (before and after FPL application) and quantification of APD at indicated % of repolarization (*n* = 5 slices of *N* = 5 male mice, *n* = 5 slices of *N* = 5 female mice; APD_50_: *p* = 0.00077, APD_80_: *p* = 0.00623, APD_90_: *p* = 0.00899). Color codes for male (black), female (red), and after FPL 64176 application (blue), respectively.

Female sex has been recognized as an independent risk factor for drug‐induced LQTS (Li et al., 2013). Ca^2+^ mishandling and resulting signaling events in cardiomyocytes, referred to as the Ca^2+^ vicious cycle, link a number of cardiac pathophysiologies with cardiac arrhythmias including LQTS (Hegyi et al., 2021), with increased Ca^2+^ influx prolonging the electrographic QT interval (Giudicessi & Ackerman, 2016). The pharmacological LTCC agonist FPL 64176 is a convenient tool to potentiate the voltage‐dependent calcium current, which balances AP repolarization (Fan & Palade, 2002), and hence afforded us to assess APD morphology under Ca^2+^ stress conditions in a sex‐dependent manner. As shown in Figure 3a, APD_80_ mapping of a representative LV epicardial region indicated that FPL 64176 (250 nM) delayed late AP repolarization in heart slice preparations from both female and male mice, stimulated at 5 Hz. However, detailed quantification of APDs after incubation with FPL 64176 demonstrates significant prolongation of medium and late repolarization by 30%–80% only in female sex, whereas there was only a tendency for a repolarization delay in male sex (Figure 3b). APD at 30% repolarization remained unaffected, irrespective of sex. These data indicate a sex‐dependent repolarization reserve becoming evident by LTCC activation.

### 3.4 Simulation indicates a dominant role for I_Na‐L_ in shaping AP repolarization under calcium stress

**FIGURE 4:**
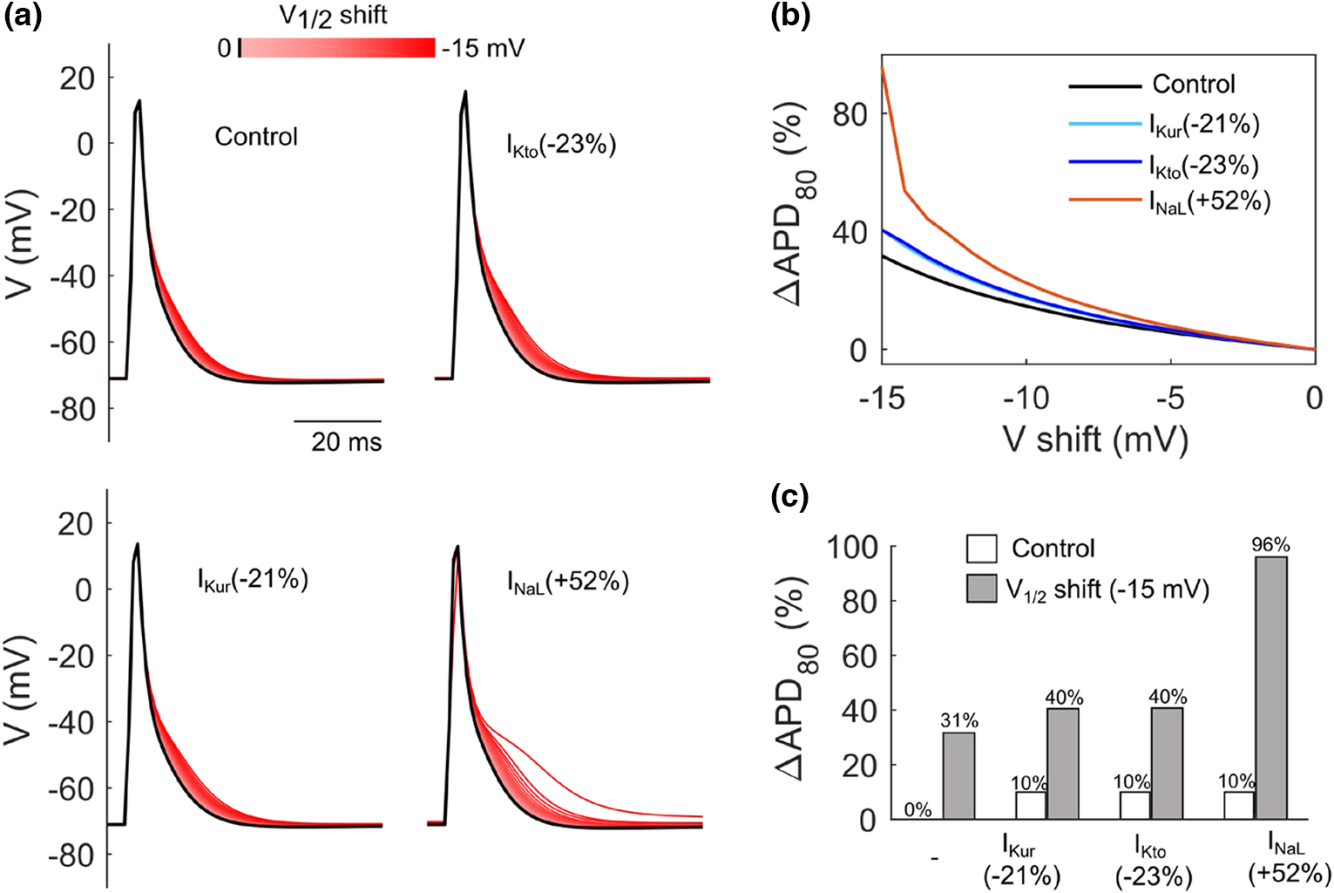
Simulation of sex‐specific contribution of ionic currents on action potential duration upon L‐type calcium channel activation. (a) Simulation of APs upon a gradual shift in voltage‐dependence of I_CaL_ activation: voltage of half‐maximum activation (V_1/2_) was shifted to negative potentials by up to 15 mV at four different conditions: control (male sex); I_Kto_ (−23%): 23% reduction in g_Kto_; I_Kur_ (−21%): 21% reduction in g_Kur_; I_NaL_ (+52%), 52% increase in rate constant (k, Figure 2c). Note the striking delay and heterogeneity in late repolarization, particularly under increasing I_NaL_. (b) Calculations of APD_80_ upon the indicated shift in voltage‐dependence of activation of I_CaL_ under corresponding sex‐specific current modifications. (c) APD_80_ alteration under indicated sex‐specific current modification with (gray) and without (white) a 15 mV shift in V_1/2_ of I_CaL_ to negative potentials.

Finally, we tried to gain more mechanistic insight into the experimentally observed sex‐dependent differences in repolarization reserve by mathematical modeling. Single mouse cardiomyocyte APs were simulated while shifting voltage‐dependence of Ca_v_ channel activation to more negative potentials (−15 mV), well mimicking the action of FPL 64176. The individual role of potassium (I_Kto_, I_Kur_) and sodium currents (I_NaL_), which had been reported to be differentially regulated in a sex‐specific manner and which were identified to contribute significantly to late AP morphology in our modeling experiments above, were assessed by adapting their respective current densities. First, for an arbitrary increase in APD_80_, the corresponding extent of current modulation was calculated. A reduction of I_Kto_ by 23%, of I_Kur_ by 21%, and an increase in I_NaL_ by 52%, was each sufficient to yield a prolongation in APD_80_ of 10% under model baseline conditions. As shown in Figure 4a, shifting voltage‐dependence of Ca_v_ channel activation by −15 mV resulted in a maximum increase in APD_80_ by 40%, which was not further affected by the additional reductions in I_Kto_ or I_Kur_ calculated above. However, the calculated increase in I_NaL_ conductivity of 52%, yielding a 10% prolongation of APD_80_ at regular Ca_v_ channel function, more than doubled APD_80_ when challenged by Ca_v_ channel overactivation. In Figure 4b, the putative current contributions to late repolarization behavior in response to a gain of Ca_v_ channel function are summarized. Taken together, mathematical modeling indicates a prominent role of I_NaL_ in shaping the late AP morphology under calcium stress.

## 4 DISCUSSION

Sex‐specific differences in cardiac AP morphology due to differential ion channel regulation are widely accepted. However, most studies on sex‐dependent expression and function of cardiac ion channels have been performed in dissociated cardiomyocytes, which do not provide the spatial context that is, on the one hand, necessary to perform correct comparisons between males and females and, on the other hand, may also contribute to functionally relevant heterogeneity between sexes (Antzelevitch & Fish, 2001; Bartos et al., 2015). Here, we demonstrate optical mapping of membrane voltage in a slice preparation of mouse heart as a feasible tool to reveal sex‐specific AP heterogeneity at high spatial resolution. By combining this approach with mathematical modeling, we were able to generate hypotheses how the observed heterogeneity in AP morphology might relate to respective single current densities in cardiomyocytes.

The present study reveals marked sex‐specific differences in AP repolarization in epicardial regions of the LV, with longer APD at late repolarization in female than in male mice. Thus, the transmural gradient in late APD observed in male mice becomes leveled in female mice, which demonstrates sex‐specific patterns of transmural heterogeneity. These findings are in good agreement with electrographic studies in humans demonstrating a larger T peak ‐T end interval in men than in women (Smetana et al., 2003). In addition, the present study highlights a strikingly higher degree of interindividual AP and APD gradient variability in female mice that may be attributed to menstrual stages, which were not controlled herein. Also, structural heterogeneity by lipid or collagen deposition may add to both intra‐ and interindividually varying ion channel expression patterns (Gerdts & Regitz‐Zagrosek, 2019). Particularly, influencing factors like hormonal state and age have always fueled the discussion about the sex‐specificity of the cardiac AP (Brouillette et al., 2003; Brouillette et al., 2005; Brunet et al., 2004; Lowe et al., 2012; Saito et al., 2009; Trépanier‐Boulay et al., 2001; Wu & Anderson, 2002; Yusifov et al., 2021). It is also noteworthy to mention that there is considerable variance between reported APD parameters in isolated cells (Figure S1, Table 2), which most likely reflects uncontrolled methodological and spatial determinants. By preserving a much more physiological cell composite in the heart slice preparation, our study convincingly confirms both longer epicardial APD and increased APD variance in female than in male mice.

The cardiac AP results from a complex voltage‐dependent interplay of a number of ionic currents de‐ and repolarizing the resting membrane potential. Thus, changes in AP morphology will reflect changes in underlying ionic currents. During recent years, extensive studies on isolated cardiomyocytes have come up with genetic or functional regulation of several candidate currents, in an attempt to explain the different features of the cardiac AP in male versus female mice; however, with numerous discrepancies in the literature. For instance, there exist several reports indicating a reduction in the ultrarapid delayed‐rectifier potassium current I_Kur_ in female mouse ventricles, which would comprehensibly prolong the APD (Brunet et al., 2004; Crump et al., 2016; Saito et al., 2009; Trépanier‐Boulay et al., 2001), whereas other studies described I_Kur_ as either indifferent from or even larger than in male mice (Brouillette et al., 2003; Brouillette et al., 2005; Wu & Anderson, 2002). As mentioned above, parameters such as the sex hormonal state or spatial context may well account for the diverging reports on sex‐dependent I_Kur_ expression. More robust, there is evidence for a smaller density of the transient outward potassium current I_to_ in females (Brunet et al., 2004; Crump et al., 2016; Saito et al., 2009); the underlying ion channel alpha‐subunits K_v_4.2 and K_V_4.3 have been shown to be less expressed in females versus males. It should be kept in mind that most ion channels assemble as multi‐protein complexes with their function being not only determined by gene or protein expression of the pore‐lining subunits, but also by auxiliary subunits and numerous post‐translational modifications affecting channel numbers on the cell surface or their gating properties (Lee et al., 2014). Intriguingly, the beta‐subunit KCNE4 modulates both I_Kur_ and I_to_ functions (Crump et al., 2016). Among the sex‐specific ionic currents may also be the late sodium current I_NaL_, which has been shown to particularly increase in female compared to male mice when specifically activated (Lowe et al., 2012). Using mathematical modeling of mouse cardiomyocyte electrophysiology, we were able to demonstrate the quantitative contribution of each of the predominant ionic currents to AP morphology. Under physiological conditions, we hypothesize I_to_ to shape both early and late repolarization, while I_Kur_ and—to a lesser extent—I_NaL_ affect only late repolarization. In contrast, the currents I_Kss_, I_Kr_, and I_Ks_ are unlikely to substantially contribute to sex‐specific AP morphology in mice, as their densities are rather negligible. As not being considered in the model, it should be kept in mind, that post‐translational regulation of these currents may indeed change their contribution (Morotti et al., 2014; Tapa et al., 2020).

We finally sought to evaluate the sex‐specific AP morphology in response to modified Ca^2+^ flux into cardiomyocytes. In a number of cardiac pathophysiologies, including hypertrophy and Long‐QT syndromes, increased Ca^2+^ influx plays a central role (Antoons et al., 2007; Hennessey et al., 2014; Wemhöner et al., 2015). Often a shift in voltage‐dependent activation of I_CaL_ to more negative potentials is observed, increasing I_CaL_ window current, which prolongs the APD and promotes the formation of early after depolarizations (Song et al., 2015). Here, we have shown that the response in AP morphology to pharmacological activation of I_CaL_ is sex‐specific, with a much more pronounced APD prolongation in females. Obviously, differential expression of I_CaL_ density may account for such observation. In female rats and dogs, cardiomyocytes exhibit higher Ca^2+^ channel densities and hence greater I_Ca_ than in cardiomyocytes from respective male animals (Saito et al., 2009; Vizgirda et al., 2002; Xiao et al., 2006). However, for mice, Johnson et al. showed that only decreased levels of estrogen are associated with an increased number of cardiac LTCCs (Johnson et al., 1997). Based on our simulation experiments increasing window I_CaL_, we assume an outstanding role for I_NaL_ in shaping the sex‐specific response of AP morphology. Among the above specified determinants of female APD prolongation in mice, I_NaL_ undoubtedly outstands in delaying APD_80_ in response to enhanced Ca^2+^ influx. Being aware of important differences in the spatial expression of cardiac ion channels between mice and humans, it suggests itself that a more prominent role of I_NaL_ in drug‐induced LQTS, which occurs more often in women than in men (Li et al., 2013), should be considered. It is worth mentioning that besides ion channels, also ion transporters may be expressed in a sex‐specific manner and hence influence the sex‐specific cardiac electrophysiology (for review: [James et al., 2007; Yang & Clancy, 2012]). Thus, at high intracellular Ca^2+^ concentrations and subsequent formation of early afterdepolarizations (EADs), a possible role of the sodium/calcium exchanger NCX has to be taken into account (Parks & Howlett, 2013).

Our study has limitations that are worth highlighting. The currently available mathematical models of single mouse ventricular myocytes show significantly shorter APDs than the ones recorded from cardiac tissue. Lacking mechanical load or mechanical uncoupling agents may contribute to this difference. Continuously revising the models based on experimental data hence appears not only necessary but will improve our quantitative understanding of sex‐specific differences in cardiac electrophysiology, eventually giving rise to sex‐specific models much better adopted to given medical needs. We selected specifically the LV epicardial region for our study, because of the significant amount of published data on sex‐specific ion channel regulation in cardiomyocytes isolated from this area and because of the availability of a respective mathematical model. Nevertheless, the herein introduced experimental approach may be generalized to other cardiac regions, including the septum and RV. Transmural slices at high spatial resolution provide even the tool to investigate sex‐specific intra‐heart APD heterogeneity. Such analyses may open new doors to understand the sex‐specific differences in cardiac arrhythmia susceptibility. Despite the present limitation of focussing on sex‐specific characterization of membrane voltage in healthy mouse heart slices, the approach is well suited to gain also comprehensive insight into sex‐specific differences in regional remodeling processes (e.g., membrane voltage and calcium signaling) after myocardial infarction and in hypertrophy. On a perspective, both superresolution microscopic and spatial omics technologies may well be combined with the herein established optical mapping and mathematical modeling approach to help test unbiased hypotheses of regulated ion channel function in both physiological and pathophysiological conditions.

## AUTHOR CONTRIBUTIONS

S. Erfan Moussavi‐Torshizi, Ehsan Amin, and Nikolaj Klöcker conceived the project. S. Erfan Moussavi‐Torshizi and Ehsan Amin performed the experiments and analyzed the data. Ehsan Amin and Nikolaj Klöcker wrote the manuscript.

## CONFLICT OF INTEREST STATEMENT

The authors declare no competing interests.

## ETHIC STATEMENT

All animal experiments comply with the EU Directive 2010/63/EU on the protection of animals used for scientific purposes. Studies were approved by the local Animal Care and Use Committee of the University of Düsseldorf (# O7/11) and the Animal Ethics Committee of the North Rhine‐Westphalia Nature, Environment and Consumer Protection Agency (LANUV).

## Supporting information


Figure S1.
Table S1.
