# Supplementary material for: Sex‐specific repolarization heterogeneity in mouse left ventricle: Optical mapping combined with mathematical modeling predict the contribution of specific ionic currents
Source: Physiol Rep. 2023 Jun 8;11(11):e15670. doi: 10.14814/phy2.15670 (PMC10250535; doi:10.14814/phy2.15670)
Supplement: Supplementary file 1 — Figure S1. Table S1. [file PHY2-11-e15670-s001.docx]

**Supplementary Figure 1:**

**
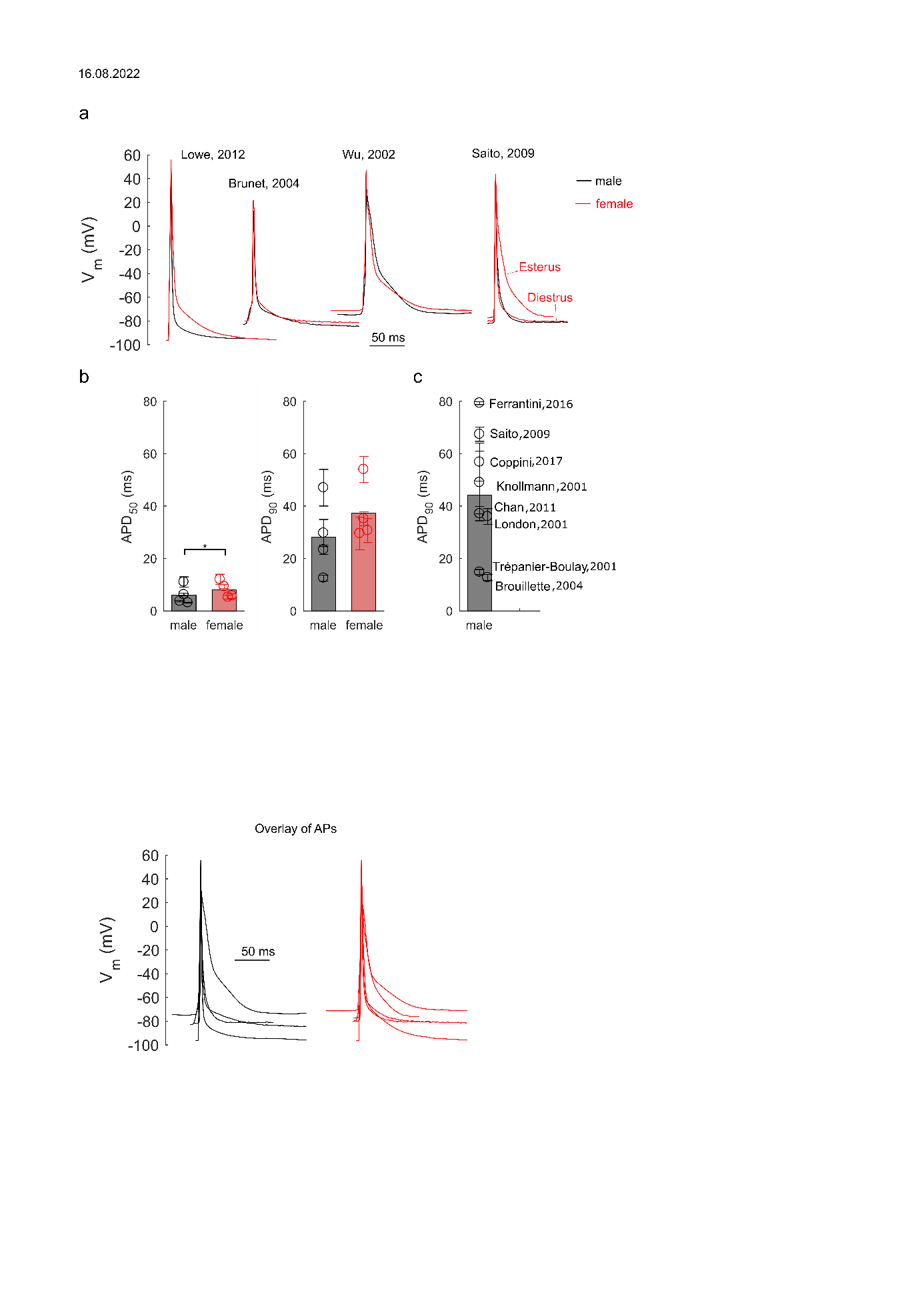
**

**Figure S1**. **Digitalization of published data on sex differences of mouse ventricle AP.** **(a)** it shows the digitalization and overlay of male/female APs from different studies. **(b)** quantification of APD_50_ and APD_90_ of APs in (a) has been depicted. **(c)** APD_90_ of individual male APs are plotted. To extract APs from literature, we digitalized the plots utilizing the online plot digitizing tool WebPlotDigitizer version 4.5 (56).

**Supplementary Tables**

**Table S1.** Estimated differences between indicated APDs and transmural gradients in male versus female mice and respective confidence intervals using hierarchical nested statistical analysis.

|  | **estimated difference (ms)** | **standard error (ms)** | **df** | **t value** | **lower limit** | **upper limit** | **Bonferroni p-value** |
| --- | --- | --- | --- | --- | --- | --- | --- |
| APD_30_ | 0.686 | 0.687 | 15.0 | 1.000 | -0.77 | 2.150 | 0.302 |
| APD_50_ | -5.744 | 3.164 | 15.6 | -1.81 | -12.46 | 0.979 | 0.080 |
| APD_80_ | -9.738 | 3.382 | 18.0 | -2.880 | -16.84 | -2.634 | 0.009 |
| APD_90_ | -9.273 | 3.024 | 18.0 | -3.067 | -15.63 | -2.920 | 0.006 |
| APD_90_ gradient | -8.292 | 1.415 | 16.8 | -5.861 | -11.28 | -5.305 | 0.00002 |
